# Physiologically Based Pharmacokinetic (PBPK) Modeling of Metabolic Pathways of Bromochloromethane in Rats

**DOI:** 10.1155/2012/629781

**Published:** 2012-04-11

**Authors:** W. S. Cuello, T. A. T. Janes, J. M. Jessee, M. A. Venecek, M. E. Sawyer, C. R. Eklund, M. V. Evans

**Affiliations:** ^1^Research Experience for Undergraduate participant, Department of Mathematics, North Carolina State University, Raleigh, NC 27695, USA; ^2^Department of Mathematics, North Carolina State University, Raleigh, NC 27695, USA; ^3^National Health and Environmental Effects Research Laboratory, US Environmental Protection Agency, Office of Research and Development, Research Triangle Park, NC 27709, USA

## Abstract

Bromochloromethane (BCM) is a volatile compound and a by-product of disinfection of water by chlorination. Physiologically based pharmacokinetic (PBPK) models are used in risk assessment applications. An updated PBPK model for BCM is generated and applied to hypotheses testing calibrated using vapor uptake data. The two different metabolic hypotheses examined are (1) a two-pathway model using both CYP2E1 and glutathione transferase enzymes and (2) a two-binding site model where metabolism can occur on one enzyme, CYP2E1. Our computer simulations show that both hypotheses describe the experimental data in a similar manner. The two pathway results were comparable to previously reported values (*V*_max⁡_ = 3.8 mg/hour, *K*_m_ = 0.35 mg/liter, and *k*_GST_ = 4.7 /hour). The two binding site results were *V*_max⁡_1__ = 3.7 mg/hour, *K*_*m*⁡_1__ = 0.3 mg/hour, CL_2_ = 0.047 liter/hour. In addition, we explore the sensitivity of different parameters for each model using our obtained optimized values.

## 1. Introduction

Bromochloromethane (BCM, CH_2_BrCl, CAS number 83847-49-8) is a volatile solvent historically used in portable fire extinguishers. BCM was gradually replaced by halons in the 1970s and banned in 2002 due to its ozone depletion potential. However, BCM is still used as an intermediate in the production of other solvents [1] and is rapidly absorbed via inhalation due to its volatility [2]. Similar to other halomethanes, there is a suggestion of hepato- and nephro-toxicity due to prolonged exposure [2]. Another potential source of exposure for BCM is through oral ingestion via water, since BCM is considered a water disinfection byproduct [3], and is listed within the U.S.A EPA's Candidate Contaminant List (CCL). Additional research on brominated disinfection by-products (DBPs) has been proposed because there is evidence that brominated species can be more potent than chlorinated compounds [4]. The occurrence of brominated disinfection by-products (e.g., bromochloromethane) is an added consideration for coastal areas due to potential bromine intrusion from seawater and subsequent transformation of chlorinated by-products into brominated species [5]. Given the potential health impact of brominated chemicals, we chose to study BCM in consideration of both increased potential for exposure near coastline utilities, and on the basis of a deficiency and availability of published data. Based on a recent PubMed search, the published literature for BCM is much less than that of the well-studied bromodichloromethane (BDCM, CHBrCl_2_, CAS number 75-27-4), another structurally related DBP.

Physiologically based pharmacokinetic (PBPK) models are computational tools that are increasingly used to address risk assessment issues, particularly in quantifying the relationship between measures of external exposure and internal dose [6]. PBPK models are sets of equations representing the flow of blood and toxicant into and out of organs and body tissues. Unlike classical pharmacokinetic models, PBPK models include species-specific physiological, chemical, and biochemical parameters, allowing for extrapolation to humans [7]. The interaction between modelers and biologists is crucial in determining the final representation of organs to ensure inclusion of target organs, special physicochemical properties and toxicity. An accurate mathematical description of biological processes (i.e., an accurate model) requires synthesis of mathematics, modeling, and systems biology. The expectation of such an approach is that an integrated modeling effort and its application to quantification of health risk assessment would increase our confidence in the simulated results.

**Figure 1:**
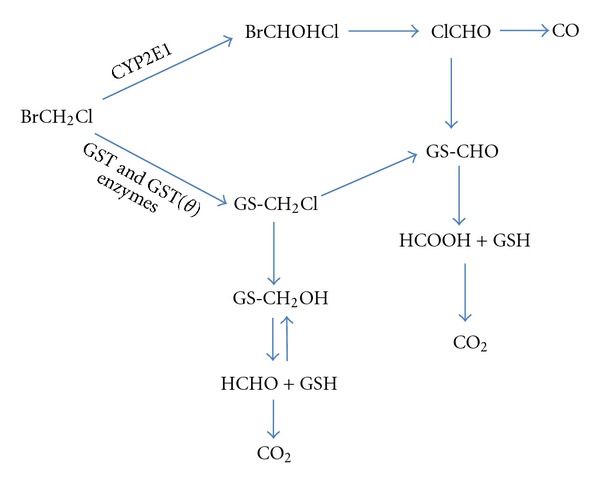
Schematic representation of BCM metabolism, adapted from [13].

For volatile halogenated methanes, metabolism is an important component studied to provide explanations of how toxicity occurs. The complex kinetics suggested for dihalomethane metabolism (e.g., BCM) consists of two components: a saturable Michaelis-Menten hyperbola superimposed with a linear pathway [9]. This linear pathway does not appear to saturate and shows a continued proportional increase at higher exposure concentrations. At lower concentrations, the contribution of this linear pathway is smaller than that of the saturable component. The question becomes what are the physiological counterparts of these two different metabolism components? What enzymes may exhibit such different characteristics? Previous PBPK modeling work with dichloromethane (DCM, CH_2_Cl_2_, CAS number 75-09-2) led to the hypothesis that two different enzymes are involved in its metabolism: CYP2E1, a P450 isoenzyme, and GST (GSH S-transferase). The early PBPK modeling work was followed by experiments showing that the GST pathway, although numerically small, was an important pathway toxicologically. Based on experimental evidence, the GST pathway is believed to be responsible for the carcinogenicity of these compounds [15]. The early PBPK modeling paved the way for establishing a two-pathway hypothesis which includes both P450 and glutathione transferase (GST) enzymes [9]. Based on these concepts, the proposed metabolic scheme for bromochloromethane is shown in (Figure 1). Specifically for BCM, the two-pathway hypothesis was first suggested by PBPK modeling followed by experimental confirmation of adduct formation from the GST pathway in bacterial assays [16]. Using an *in vitro* Salmonella assay, BCM was confirmed to be mutagenic in the presence of cytosol (GST containing fraction) relative to other structurally related halomethanes [17]. Based on structural similarity with other brominated ethanes (i.e., 1,2-dibromoethane), where plasma bromide was used to track metabolism, the existence of a small but relevant GST pathway is to be expected for BCM [18]. However, we were not able to find *in vivo* studies that can be used to quantify the linear pathway for BCM, other than suggestions that *in vivo* data is lacking [13].

**Figure 2:**
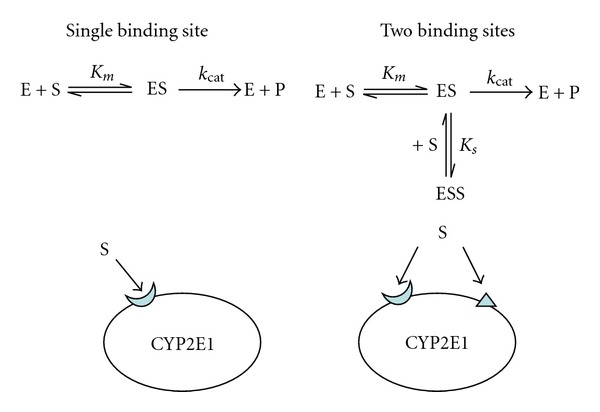
Comparison of single-sited and two-sited kinetics, adapted from [14].

Other than having two different enzymes describe the complex kinetics, is there another plausible explanation for the linear kinetics exhibited by the *in vivo* closed chamber data? If so, can PBPK modeling be used to explain the additional linear kinetics? In recent years, the realization has been made that several P450s may exhibit atypical kinetics, that is, complex kinetics that cannot be explained by the single saturable Michaelis-Menten kinetics [19]. In an attempt to better describe these increased complexities, biochemical models with two sites have been developed to describe the linear kinetics exhibited at higher concentrations [20]. These complex biochemical models have an additional binding site, which is simultaneously available to the substrate (Figure 2). Once binding to the second site occurs, a conformational change takes place that modifies the metabolism of the first site. We propose that a PBPK model for BCM can be constructed to show the plausibility of a second site model. At this point in time, we are not suggesting that one kinetic model is more realistic than the other, but are investigating the possibility of alternative pathways based on atypical kinetics and its implications for metabolism. Interestingly, one potential way to distinguish between the two mechanism is by the type of metabolites excreted. With two-sites in a single enzyme, the metabolites produced would probably be reactive products of CYP2E1 oxidation. In contrast, a two-pathway mechanisms would have glutathione containing metabolites during excretion.

When testing a new hypothesis using PBPK model simulations, one possible question becomes how to rank the importance of relative metabolic parameters. We believe that this question is particularly relevant when trying to distinguish between metabolic constants that are numerically small. Sensitivity analysis is a mathematical tool that allows determination of how the change in one parameter affects model predictions. When conducting sensitivity analysis, the model is run repeatedly to determine how changes in parameter values relate to changes in a prediction. Although optimization is performed before sensitivity analysis, modeling behavior insights come from the sequential sensitivity analysis. The sensitivity analysis results will also be dependent on the values obtained from the optimization. Both techniques are combined since the first is needed to find the “correct” parameter values, while sensitivity analysis looks for impact across parameters. In the present work, both techniques were used to identify potential issues with metabolic parameter quantification. Once the optimization is performed, sensitivity analysis can determine if a unique set of values exist for the optimized results. In general, sensitivity analysis has been used in combination with PBPK modeling to estimate the relative ranking of different model parameters [21], sometimes including the interaction between parameters [22]. Local sensitivity analysis is the simplest form of sensitivity analysis, which allows for estimation of the impact of each parameter individually. One potential analogy is that this type of sensitivity analysis is similar to a worst-case scenario estimate for the variability of each parameter. In addition to ease of implementation, another advantage of this analysis is its ability to sort potential identifiability issues, or to state whether the optimization provides unique estimates for the resulting values obtained for the different modeling hypotheses.

In summary, the goals of this study were (1) to use PBPK modeling in combination with BCM closed chamber data to test two potential metabolic hypotheses: a two-pathway enzyme combination of CYP2E1 and GST and a one enzyme mechanism consisting of two binding sites for CYP2E1; (2) to make use of sensitivity analysis to identify important model parameters; (3) to compare the fits across different metabolic hypotheses and suggest future experiments that lead to increased confidence in our simulations.

## 2. Methods

### 2.1. PBPK Models

#### 2.1.1. Closed Chamber Data

Inhalation closed chamber data for BCM in rats were obtained from the published literature [9] digitized using UN-SCAN-IT 6.0 (Silk Scientific, Orem, UT, USA). Inhalation closed chamber data were collected from a sealed system, allowing for quantification of metabolism. A BCM bolus was injected into the chamber at time zero. After equilibrium between the rat and air inside the chamber, BCM decreased with time as it was distributed into tissues and metabolized by the rodent. A PBPK model was then constructed to describe the decline in air chamber concentration. The metabolic parameters were calculated using optimization techniques in combination with the inhalation data. Different metabolic hypotheses were investigated by using a PBPK model with the closed chamber-derived metabolic parameters to predict metabolism.

#### 2.1.2. Core Model

**Figure 3:**
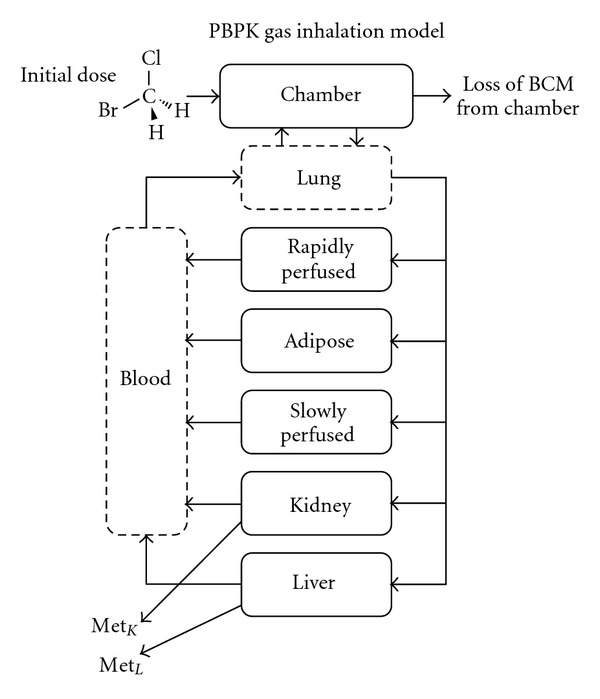
Schematic representation of PBPK model used for BCM.

A flow-limited PBPK model based on the structure described by Ramsey and Andersen [23] was used to describe BCM metabolism (Figure 3). A seven-compartment model (lung, blood, adipose, rapidly perfused tissue, slowly perfused tissue, liver, and kidney) was developed for BCM, with the assumption that metabolism occurred via the same enzymes in liver and kidney. All physiological parameters were obtained from the literature as shown in Table 1. Differential equations based on mass conservation principles were derived for each organ. Lung and blood compartments use a steady state assumption, simplifying the overall solutions for the model. Tissue to blood partition coefficients were calculated using experimentally-derived BCM coefficients from Gargas et al., 1986 [9] (described in the appendix). After derivation, all equations were coded into MatLab (MathWorks, version 7.11.0.584) using the equation solver *ode15s*.

#### 2.1.3. Metabolic Hypotheses Testing

The current work tested two different metabolic hypotheses using the same basic PBPK model structure, varying only the equations for liver and kidney metabolism. Previously, Gargas et al. [9] evaluated typical Michaelis-Menten kinetics, with metabolism described by a single CYP2E1 binding site. The results of their simulations demonstrated that the addition of a linear term was necessary to describe the closed chamber data. Thus, a two-pathway model with two different enzymes, CYP2E1 exhibiting Michaelis-Menten kinetics and GST exhibiting linear kinetics, provided much better fits to the data than the model that incorporated only Michaelis-Menten kinetics [9]. Here, the two-pathway model (Michaelis-Menten and GST) described by [9] is compared to the two-binding site model described by Evans and Caldwell [24]. In the two-binding site model, a modified Michaelis-Menten equation that includes two binding sites for CYP2E1 is used to describe BCM metabolism.

#### 2.1.4. Two-Pathway Kinetics

The two-pathway description uses Michaelis-Menten kinetics in addition to a linear term to account for GST metabolism. The equations listed below represent the additional metabolic term in both the liver (*Met*_*l*_) and the kidney (*Met*_*k*_). Liver is set to account for 94.8% of total body metabolism and the kidney 5.2% based on [25]:

(1) $$\begin{array}{cc} Met_{l} & =0.948\left(\frac{V_{\max}\left[{\text{C}}_{\text{liv}}\right]}{K_{m}+\left[C_{\text{liv}}\right]}+k_{\text{GST}}\left[C_{\text{liv}}\right]V_{\text{liv}}\right),\\ Met_{k} & =0.052\left(\frac{V_{\max}\left[C_{\text{kid}}\right]}{K_{m}+\left[C_{\text{kid}}\right]}+k_{\text{GST}}\left[C_{\text{kid}}\right]V_{\text{kid}}\right), \end{array}$$

where (i) *V*_max⁡_: maximum velocity of the reaction, mg/hour, (ii) *K*_*m*_: affinity constant, mg/liter, (iii) *k*_GST_: proportionality constant for linear pathway metabolized by glutathione transferase, /hour.

#### 2.1.5. Two-Binding Site Kinetics

Linear kinetics at higher concentrations can also be described using a dual binding site, as demonstrated for dichloromethane using closed chamber data [24]. In this case, CYP2E1 undergoes a structural change in which two separate binding sites are simultaneously available to BCM, and metabolism is achieved with one enzyme. For the dual binding site equation, the squared concentration term becomes asymptotically linear at higher exposure concentrations. This squared term is not present in the typical Michaelis-Menten equation and will lead to a different rate of ascent to the asymptote. The modified Michaelis-Menten equation reflecting two binding sites in one enzyme [20] becomes

(2) $$\begin{array}{c} \begin{array}{cc} Met_{l} & =0.948\left(\frac{V_{\text{ma}{\text{x}}_{\text{1}}}\left[{\text{C}}_{\text{liv}}\right]+\text{C}{\text{L}}_{2}{\left[C_{\text{liv}}\right]}^{2}}{K_{m_{1}}+\left[C_{\text{liv}}\right]}\right), \end{array}\\ \begin{array}{c} \begin{array}{cc} Met_{k} & =0.052\left(\frac{V_{\text{ma}{\text{x}}_{\text{1}}}\left[C_{\text{kid}}\right]+\text{C}{\text{L}}_{2}{\left[C_{\text{kid}}\right]}^{2}}{K_{m_{1}}+\left[C_{\text{kid}}\right]}\right), \end{array} \end{array} \end{array}$$

where (i) *V*_max_1__: maximum metabolic rate for the first binding site, mg/hour. The second binding site is accounted for by CL_2_, (ii) *K*_*m*_1__: affinity constant for the first binding site, mg/liter, (iii) CL_2_: clearance constant consisting of the ratio of the maximum metabolic rate and the affinity constant, *V*_max_2__/*K*_*m*_2__, for second binding site, liter/hour.

### 2.2. Comparison of the Different Metabolic Models

A total of six metabolic parameters were optimized using five closed chamber data sets, each having a different starting concentration. Optimizations were performed using the natural log of the data with MatLab's *fminsearch* function (MathWorks, version 7.11.0.584). For the two-pathway model, three parameters were optimized using the digitized data: *V*_max⁡_, *K*_*m*_, and *k*_GST_. For the two-binding site model, three parameters were optimized using the digitized data: *V*_max_1__, *K*_*m*_1__, and CL_2_. The function *fminsearch*, available in the standard MatLab package, tries to minimize a “cost” as the minimum difference between data and simulation. The best overall fit minimized by *fminsearch* includes the entire data range (200–4000 ppm).

### 2.3. Physiological Value Selection

The physiological values to be used in the PBPK model have been updated from those used in the original simulations of the data in 1986. As an example, cardiac output values for F344 rats are now available [11]. The range of normalized cardiac output values for F344 rats values (QCC) is between 10–20 liters/hour/kg^0.75^, due to dependence of cardiac output on age. We decided to use the mean value of 15 liters/hour/kg^0.75^, since a 225 g rat is considered adult [10]. The ventilation rate for rats was calculated using the ventilation perfusion ratio, which varies between 1–4 fold due to differences in measurement methodology [22, Table 31]. Since the mean value of 15 liters/hour/kg^0.75^ matched the values used in the benzene PBPK model dated from approximately the same time period as the Gargas dataset, we used the same ventilation perfusion rate as in Medinsky et al. [12] for consistency. The F344-specific tissue perfusion and volume values were adopted from [11].

### 2.4. Sensitivity Analysis

Sensitivity coefficients were calculated with partial derivatives for each variable of interest with respect to the model parameter being specified in the model. Sensitivity coefficients were then normalized by both the variable and model parameter. The resulting time course for each sensitivity coefficient was plotted. Sensitivity coefficients were calculated using Automated Differentiation written by Martin Fink and available at MatlabCentral(http://www.mathworks.com/matlabcentral/). Another Matlab function written by Adam Attarian (*tssolve* NC State University, Raleigh, NC, USA) was used to organize the partial derivatives and for the final plots. A three-dimensional surface was generated to show variation in sensitivity coefficients with time, and variability within the metabolic parameter. These tests were performed to check stability of the sensitivity coefficients when introducing variability around the optimized estimates.

## 3. Results

**Figure 4:**
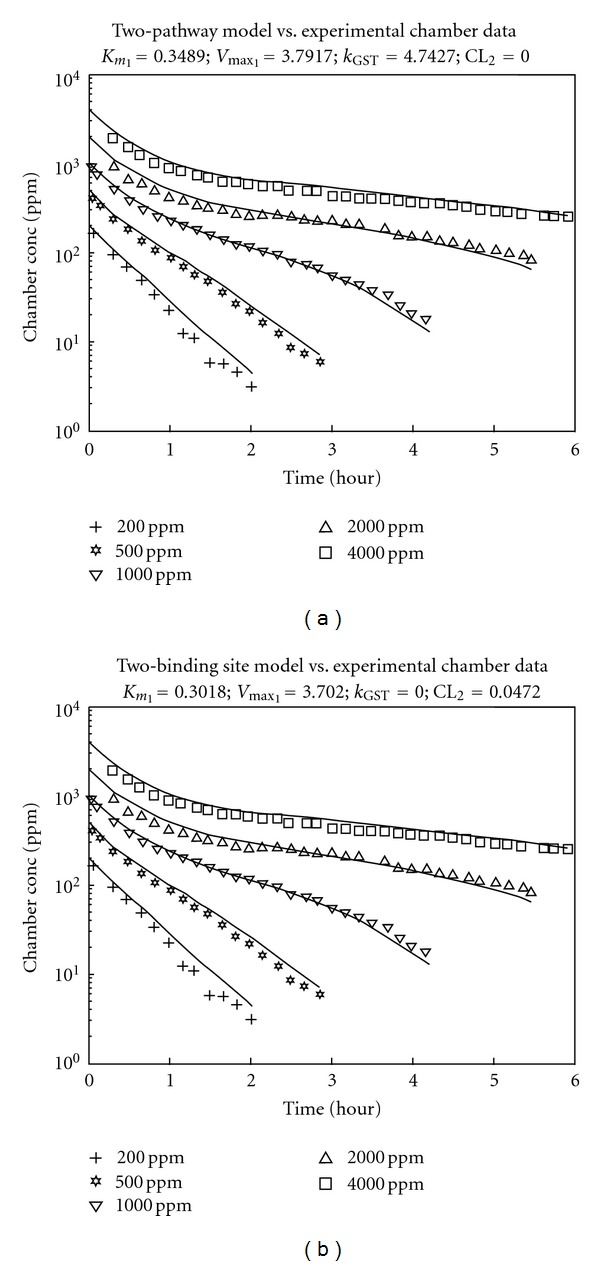
PBPK modeling results using two different metabolic hypotheses. (a) shows results for the two-pathway model. (b) shows results for the two-binding site model.

The optimization results are shown in Table 2. For all concentrations studied, the two-pathway and two-binding site models gave very similar descriptions of the closed chamber data (Figure 4). Both the *V*_max⁡_ and *K*_*m*_ values are similar for the two-pathway and two-binding models. The two-pathway parameter values were as follows:

(3) $$\begin{array}{c} V_{\max}=3.8\;\text{mg}/\text{hour},\\ K_{m}=0.35\;\text{mg}/\text{liter,}\\ k_{\text{GST}}=4.7/\text{hour}. \end{array}$$

The two-binding site hypothesis uses Michaelis-Menten parameters *V*_max⁡_1__ and *K*_*m*_1__ to describe the first site, and clearance CL_2_, defined to be the ratio of *V*_max⁡_2__ and *K*_*m*_2__, for the second site. The two-binding site results were as follows:

(4) $$\begin{array}{c} V_{\text{ma}{\text{x}}_{\text{1}}}=3.7\;\text{mg}/\text{hour},\\ K_{m_{1}}=0.3\;\text{mg}/\text{liter,}\\ \text{C}{\text{L}}_{\text{2}}=0.047\;\text{liters}/\text{hour}. \end{array}$$

When comparing the values for both metabolic hypotheses, Michaelis-Menten results for the two-binding site hypothesis are very similar to the Michaelis-Menten values for the two-pathway hypothesis. The *V*_max⁡_ and *K*_*m*_ values are similar for the two pathway and two-binding models. Based on these results, both metabolic hypotheses are considered equally plausible.

**Figure 5:**
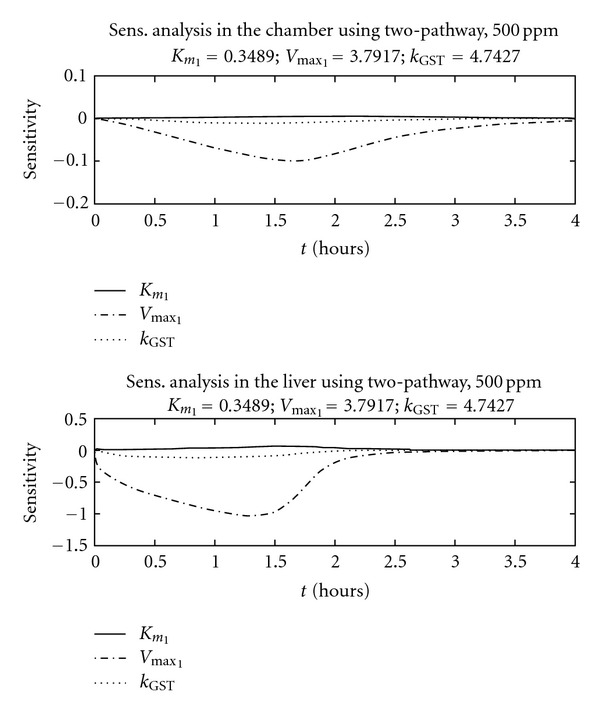
Sensitivity coefficients for the two-pathway model at 500 ppm. Each concentration includes the analysis using air and liver concentrations.

**Figure 6:**
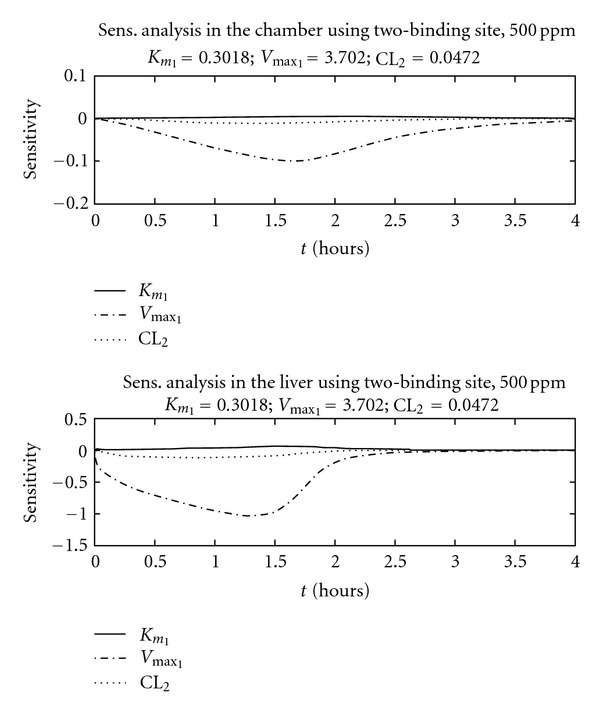
Sensitivity coefficients for the two-binding site model at 500 ppm. Each concentration includes the analysis using air and liver concentrations.

Normalized sensitivity coefficients for all model parameters were calculated after optimization results were obtained. The majority of resulting sensitivity coefficients were not shown, since we are focusing on metabolic parameters: *V*_max⁡_, *K*_*m*_, *k*_GST_, or CL_2_. The authors performed sensitivity analysis using 500 ppm as the initial concentration. Each sensitivity figure consists of two panels. The first panel presents sensitivity coefficients calculated for the impact of air chamber concentration on the simulated value of *V*_max⁡_ and the second panel presents sensitivity coefficients for liver concentration of BCM.  Figure 5 presents sensitivity results for the two-pathway model. The sensitivity coefficients for *V*_max⁡_ have the highest values. The *V*_max⁡_ sensitivity coefficients for liver concentration are larger than those obtained using chamber air as the experimental variable.  Figure 6 presents sensitivity results for the two-binding site model. The sensitivity coefficients for *V*_max⁡_ also have the highest values, particularly when comparing liver coefficients versus chamber air coefficients. For both model hypotheses, peak *V*_max⁡_ sensitivity occurs within the first two hours.

**Figure 7:**
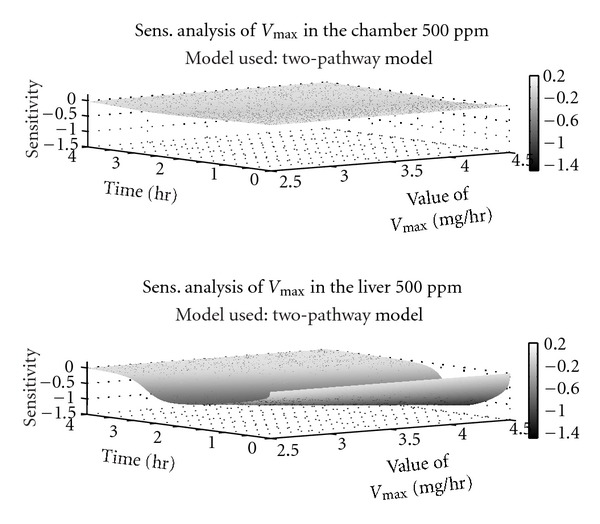
Three-dimensional sensitivity analysis plot for the two-pathway model at 500 ppm.

A three-dimensional plot of liver sensitivity coefficients for the two-pathway model is presented in Figure 7. The third dimension represents variability in *V*_max⁡_ generated by repeating sensitivity analysis simulations that vary *V*_max⁡_ between 2.5–4.5 mg/hour (to include the mean value 3.8 mg/hour). Based on the value of *V*_max⁡_, there is a peak in sensitivity to *V*_max⁡_ occurring within the first 3 hours. The chamber air concentration plot shows a much shallower surface, with no obvious peaks in sensitivity towards *V*_max⁡_ shown.

## 4. Discussion

Risk assessments for volatile chemicals have used PBPK models to account for differences across routes and extrapolate from rodent experiments to human populations [26, 27]. The use of a PBPK model allows for the integration of physiological, chemical, and biochemical information with different kinetic hypotheses. Closed chamber inhalation data are a measure of total rate of metabolism represented by the decrease in the concentration of the volatile chemical inside the chamber after a bolus injection. One application of closed chamber inhalation data in combination with PBPK modeling has been the suggestion of metabolic hypotheses leading to effects in a target organ, particularly for halomethanes [9, 28]. In this context, PBPK models have been proposed as useful computational tools for risk assessments.

PBPK models have played a decisive role in the suggestion of an *in vivo* role for the GST pathway (two-pathway model) and its contribution to toxicity for halogenated compounds [15, 28]. In the case of DCM, the GSH conjugation pathway has a small proportionality constant (*~*0.01/hour [24]), while the GST pathway for BCM is larger (*~*5.3/hour [9]). Following the inclusion of a linear pathway in the PBPK model for halogens, *in vitro* work in bacterial systems provided the first evidence that GST was the enzyme associated with the additional linear pathway. *In vitro* work also suggested that as the number of bromines included in the compound increased, the resulting genotoxicity was larger. For example, Thier et al. [16] demonstrated that the genotoxicity of CH_2_Cl_2_ (DCM) was less than that of CH_2_ClBr (BCM), which was less than that of CH_2_Br_2_ (dibromomethane) toxicity. Prior to these experiments, there was no experimental evidence that dihalomethanes such as BCM were metabolized via a GST pathway. The only suggestion for the existence of this second pathway came from PBPK modeling and closed chamber data.

The current PBPK model making use of a two-pathway mechanism gives a similar *k*_GST_ value to that of previous models (Table 2 versus [9, 29, 30]). (It is important to note that both PBPK models in [29, 30] were based on [9]. In addition, the *V*_max⁡_ and *K*_*m*_ values are slightly different when compared to [9]: 2.57 mg/hour and 0.3 mg/liter, resp.). We attribute the difference in optimized values to differences in physiological constants used in our model. The current PBPK model made use of F344 specific values [11], which correlates to the strain used in the closed chamber experiments. It is important to note that the F344 physiological values were not available at the time when the first BCM model was published (1986). The largest differences in volume or flow for the compartments used in the two different BCM PBPK models probably lie in the lumped compartments, namely, the rapidly and slowly perfused compartments.

**Figure 8:**
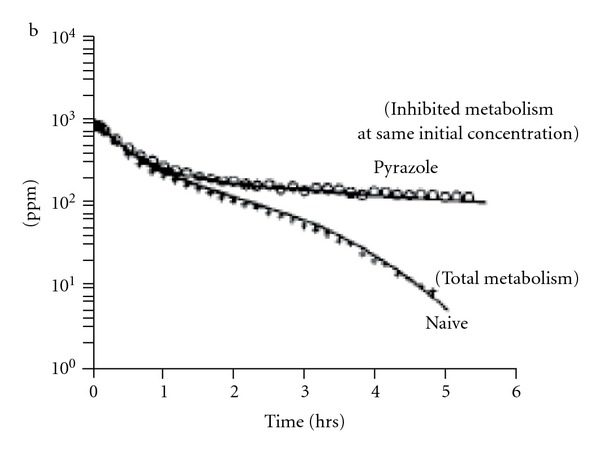
BCM vapor uptake with pyrazole treatment [9].

To our knowledge, there is no previous PBPK work that makes use of a two-binding site mechanism for BCM, making a comparison of our linear CL_2_ constant with previous models not possible. An important realization of this PBPK modeling effort is that both metabolic hypotheses appeared equally plausible. In order to help convince ourselves that this was the case, the available BCM *in vivo* literature was studied. Early *in vivo* experiments with halomethanes confirmed the enzymatic role for GST for other iodinated or brominated compounds but not for BCM [31]. This paper refers to earlier work and states that specifically for BCM, a BCM-GST reaction is not present in measurable amounts. This early indication concludes that GST is not involved as a major component of BCM metabolism. Another indication that the GST pathway is very small *in vivo* comes from the vapor uptake experiments, where treatment with pyrazole almost completely suppressed BCM metabolism as shown in Figure 8, from [9]. Since CYP2E1 has been shown to be the major P450 contributing to its oxidative metabolism [32], the *in vivo* metabolic decrease observed at this high concentration argues for the predominance of CYP2E1 over other P450s. In addition, the *in vitro* kinetic values reported by [16] also suggest a small kinetic constant when compared to ethylene dibromide or dibromomethane. The ability to numerically estimate metabolic constants for the linear pathway using sensitivity analysis will be discussed later.

Several P450s (2A1, 2B1, and 2E1) have shown atypical kinetics, explained by allosteric mechanisms making use of a second site at least *in vitro* [33]. Li et al. [33] performed simulations to confirm the relative small size of CYP2E1's active site, stating that for this enzyme the reaction proceeds by the cooperative binding of two substrates simultaneously to form a ternary complex. The substrate that binds in the active site is usually called active substrate, while the other one that binds in the effector site, and that controls the oxidation of the active substrate is called effector substrate [33].

Effector substrates are becoming increasingly studied to offer potential explanations for drug-to-drug interactions. CYP2E1 specifically plays a diverse role in physiology, toxicity, and metabolism. For example, CYP2E1 is a P450 isoform with an important role in glycogenesis, and its physiological role is increasingly being recognized [34]. At present, this isoform is known to metabolize about 70 different compounds of varying size, including alcohols, ketones, nitrosamines, anesthetics, and even long-chain fatty acids. One of the questions recently resolved by studying human CYP2E1 structure is based on addressing how is it possible for this enzyme, with a small active site, to metabolize substrates ranging in a wide number of sizes (including fatty acids). In fact, it is the suggestion of a channel structure available as a second site, and in proximity to the active site, that becomes a solution to help explain CYP2E1's flexibility to accommodate different sized substrates [35]. Although BCM is a small molecule, probably able to fit in the active site, the existence of a second site does describe linear kinetics at higher exposure concentrations. The second site model thus became a candidate for inclusion in a PBPK model calibrated using *in vivo* data for BCM. Since the second binding site in CYP2E1 is involved in fatty acid metabolism, we propose that BCM binding to this second site may impair fatty acid metabolism and may indirectly prevent normal metabolism of larger molecules that may be already impaired in chronic illnesses such as diabetes. This proposed effect is hypothesized to occur in addition to the glutathione-based toxicity based on a two-pathway hypothesis. 

When using optimization to estimate metabolic model parameters, correlation can be an important issue that may impact the ability to estimate unique values for the parameters. Basic algebra reminds us that a system of equations can be uniquely solved when the number of parameters is equal to the number of equations, assuming that the estimates are independent of each other. In general, the unique estimation of *K*_*m*_ will depend on having an independent estimate for *V*_max⁡_. Since *K*_*m*_ is interrelated to *V*_max⁡_, experiments with multiple concentrations are needed, and the concentrations used must be high enough that metabolic saturation is reached. 

One of the applications of local sensitivity analysis is to help illustrate the existence of these potential correlations between parameters because this affects the ability to solve for unique estimates. For more complex metabolic models, the relationship between metabolic parameters may not be obvious. Sensitivity analysis theory states that parameters are uniquely identifiable when the sensitivity coefficients cannot add to zero [36]. For example, if all sensitivity coefficients examined are positive, then their sum cannot be equal to zero. Using this reasoning, these authors examined the time course plots for 500 ppm and the sensitivity coefficients for both metabolic hypotheses. The sensitivity coefficients can be estimated for the different variables included in the model, allowing the analysis to suggest improvements in the experimental design. For this reason, liver concentration was selected as a possible experimental variable. When compared with chamber concentration, liver concentration resulted in increased sensitivity towards *V*_max⁡_. Since sensitivity coefficients for other metabolic parameters were nearly zero, *V*_max⁡_ is uniquely identifiable when using closed chamber experiments. At higher concentrations, the sensitivity coefficients for both *k*_GST_ or CL_2_ increase with exposure time (results not shown). This current sensitivity analysis corroborates the importance of these two additional parameters at higher concentrations.

We utilized a method using local sensitivity analysis as an initial step to help determine identifiability issues. When unique identifiability is guaranteed, the ability to have one unique solution is also guaranteed. Therefore, local sensitivity analysis can be seen as a first step towards increasing our confidence in the parameters obtained as a solution. The results of our sensitivity analysis suggest that measuring liver concentration would provide improved estimates for *V*_max⁡_ at intermediate concentrations and improved estimates for the linear constants *k*_GST_ or CL_2_ at high concentrations. To combat uncertainty in the estimates for *V*_max⁡_ and other metabolic parameters, we implemented a novel approach by estimating the three-dimensional surface for the sensitivity coefficient changing with time, and including variability in the metabolic estimate of concern. If future experiments include liver concentration measurements, our three-dimensional analysis at 500 ppm suggests that peak information on *V*_max⁡_ occurs before 2 hours, suggesting that experiment duration can be shortened by several hours. The application of PBPK modeling and three-dimensional sensitivity analyses can be helpful to design future experiments aimed at refining metabolic estimates.

The combination of optimization and sensitivity analysis presented in this paper also leads to different suggestions for future experimental design. *In vivo* experiments using the vapor uptake approach take advantage of a non-invasive approach to estimate *V*_max⁡_ and *K*_*m*_ by using air chamber concentration. However, the current sensitivity analysis suggests an increased advantage to using *in vitro* approaches to estimate metabolic parameters. The sensitivity analysis performed consistently indicates that liver tissue measurements increase our ability to estimate *V*_max⁡_ and *k*_GST_ for the two-pathway hypothesis. The same sensitivity analysis tools also indicate that our ability to estimate *V*_max_1__ and CL_2_ for two-binding site hypothesis is similar to that for the two-pathway hypothesis. The sensitivity analysis for liver tissue concentration also suggests a peak or maximum ability to estimate *V*_max⁡_ for an experiment taking 2 hours (instead of 6). Future experiments can include the possible combination of *in vivo* and *in vitro* vapor uptake techniques performed using intermediate concentrations just above and below metabolic saturation (500 ppm in this case). 

Future *in vivo* research is needed to answer definitely the question as to whether BCM's atypical metabolism can be described by a two different enzymes or by one enzyme with multiple binding sites. In this paper, we have used PBPK modeling to demonstrate the plausibility of both mechanisms in describing a linear kinetic pathway at higher concentrations. In order to determine if two separate enzymes are involved, a pharmacological agent can be used to deplete the GST pathway to determine if metabolism can proceed without GSH conjugation. As an example, such experiments have been performed for dichloromethane, using phorone as an pharmacological agent leading to GSH depletion [37]. These experiments were performed *in vitro*, using microsomes harvested from the GSH-depleted animals and used with vial equilibration techniques (to determine total changes in amount metabolized *in vitro*). Based on their results, the GST pathway was a small quantitative component of the total amount metabolized. Based on calculations provided by the current PBPK model, the GST pathway is expected to be less than 5% of the total amount metabolized. This small percent of amount metabolized difference is what is being described as either GST- or CYP2E1-dependent. In order to experimentally measure the difference in GST-mediated metabolism, a pharmacological agent such as phorone could be used for the next series of closed-chamber experiments and compared to BCM exposure without the phorone exposure. The difference between the two experiments would provide the actual difference in total metabolism observed between the two pathways.

As stated before, closed chamber inhalation experiments quantify changes in total chemical disappearance; thus, additional experiments are necessary to identify the actual P450 isoform involved in BCM metabolism. The confirmation of CYP2E1 as being the major P450 involved for *in vivo* BCM metabolism was obtained by using different P450 specific inhibitors and inducers to examine differences in CO production [32]. The use of inhibitors has become an important experimental tool to discern kinetic mechanisms involved in metabolism of different P450 isoforms. Pyrazole (CAS number 288-13-1), and its derivatives have been, used as CYP2E1 inhibitors by measuring total metabolic disappearance in microsomal fractions [38]. Recently, a structurally analog of pyrazole, 4-methylpyrazole or (4-MP), has been used to describe its CYP2E1 inhibition properties using a second-site kinetic mechanism [14]. These authors explained the inhibitory mechanism for 4-MP by adding a second-site to fit the experimental results using pNP (4-nitrophenol, CAS number 100-02-7) oxidation as a marker for CYP2E1 activity [39]. Future *in vivo* closed chamber experiments can make use of the *in vitro* paradigm used by [14] in that increasing pyrazole concentrations can be used to distinguish between the single-site or double-site models. These proposed experiments would add a dose response element to the single dose pyrazole inhibition experiments already performed by [9] as shown in Figure 8. A PBPK model can then be used to describe the resulting pyrazole inhibition as either containing a single or double site enzyme.

In summary, a combination of closed chamber data and PBPK modeling was used to examine different metabolic descriptions for BCM. The standard two-pathway description was compared to a two-binding site model within the same enzyme. Metabolic parameters for the different descriptions were optimized using the gas uptake data for BCM. Different metabolic parameters have different concentration ranges that determine their ability to be measured, and sensitivity analysis was used to demonstrate identifiability relationships between parameters. The benefits of these computational tools towards improved health risk assessments rely on their ability to accurately describe metabolic hypotheses.

## 5. MatLab Codes

There are three distinct sections to the code used to generate the results found in this paper. These sections are Data Transformations and Initialization, Compartmental Models, and Sensitivity Analysis. In addition to the brief descriptions below, each function has an extensive help section at the top of each *m* file. Note that the names of the functions are italicized for clarity.

### 5.1. Data Transformations and Initialization

Almost all of the codes used for modeling require access to physiological parameters and the data set; the script file *params* writes these parameters into the working directory as well as converts the original data (in parts per million) to a form usable by the model (in mg/liter). Note that *params* is called before any other function as it generates a. *mat* file containing relevant parameters called by the other functions in the model.

### 5.2. Compartmental Models

 The heart of the compartmental model is *ode15s*, a stiff ordinary differential equation (ODE) solver part of the standard MatLab package. There are several functions that build upon the output from the ODE solver. The main file, *BCMmain*, allows the user to choose to optimize for the metabolic parameters or to use the inputted values to plot the original data concentrations (in ppm) versus the model curves on a logarithmic scale.

*BCMmain*, when used strictly as a plotting tool, is self-contained (aside from calling the file generated by *params* and the ODE solver). In addition to a plot output, *BCMmain* also outputs a string containing the absolute root mean square error (rmse) and the relative rmse for the fit of each model at each concentration. The absolute error is calculated by comparing the two-norm of the vector containing the difference between the model predictions and the given data divided by the number of elements in the vector. The relative rmse error is given by the absolute error at each concentration divided by the initial concentration. This gives a sense of the percent error in the model.

When choosing to optimize for metabolic parameters, *BCMmain* calls on the function *optthis*, which uses a combination of *fminsearch* and a least-squares method to optimize for the parameters. The function *fminsearch*, available in the standard MatLab package, tries to minimize a “cost”; we define this cost to be the sum of the difference (obtained by linear-least squares) between the model predictions and data at each concentration. The equations for the PBPK model are contained in the file *pbpk*, which is called upon by the ODE solver and the curve-fitting functions. The output of *optthis* is the vector of metabolic parameters that yields the best fit (after a specified number of iterations) used to plot the model in *BCMmain*.

### 5.3. Sensitivity Analysis

There are two main functions used for the sensitivity analysis aspect of the code. The first function, *plot2dsens* takes in user-specified parameters and plots the sensitivity of these parameters in the chamber and the liver compartments. We choose these compartments due to their importance in obtaining data from the chamber as a noninvasive way; however data obtained from the liver may yield more insight. A second function, *plot3dsens*, and its associated helper function *modelchoice*, are designed to give a three-dimensional plot that shows the sensitivity of one parameter with respect to time as it varies over a user-input range in each compartment of interest. This analysis divides up the range of the parameter of interest into a user-specified number of subintervals and calculates the sensitivity of the parameter using the subinterval value as the parameter value in the same sense as the two-dimensional model. The sensitivity plots are then spliced together into a three-dimensional plot.

Both sensitivity functions require the use of *tssolve*, created by Adam Attarian (North Carolina State University, http://www4.ncsu.edu/~arattari/), which in turn requires Martin Fink's *myAD* package. Both functions are available from the MathWorks website (http://www.mathworks.com).

### 5.4. Code Evaluation

One of the questions addressed in the present work is how to validate new code that is generated to study a chemical, such as the case for BCM. These authors approached this question on several fronts. First, a search was made for existing code using a similar chemical to BCM. The previously published DCM model [24] was selected for a comparison with BCM. The DCM code was converted to model BCM by changing chemical-specific parameters to reflect those of BCM. The simulations were performed using the BCM/DCM version. Results of these simulations were then compared with the newly created BCM code and found to be identical. Once the new BCM code was verified to be correct, the second step was to optimize for the parameters describing two-pathway kinetics and determine their similarity with previously published values (see Results). This additional step ensures that the new PBPK model description accurately depicts BCM metabolism.

## Figures and Tables

**Table 1:** Parameters for adult Fisher 344 rats and corresponding significance in the PBPK model. (Note: partition coefficients are unitless [8].)

Parameter		Significance	Value	Source
Miscellaneous	*BW*	Body weight	0.25 kg	[9]
	*QC* *C*	Cardiac output	15 L/hr/kg^0.75^	[10–12]
	loss	Chamber loss rate	0.025 (1/hr)	[9]
	*N*	Number of rats	3	[9]
	*V*_cha_	Chamber volume	9 (L)	[9]

Flow rates (L/hr)	*QV*	Ventilation/perfusion rate	1.7	[12]
	*q*_1_	Rapidly perfused blood flow rate	*QV* − ∑*q*_*i*_	∑*q*_*i*_ is the sum of other compartments
	*q*_2_	Blood flow fraction to adipose	0.082	[11]
	*q*_3_	Blood flow fraction to slowly perfused	0.257	Muscle+bone+skin values from [11]
	*q*_4_	Blood flow fraction to kidney	0.138	[11]
	*q*_5_	Blood flow fraction to liver	0.242	[11]

Partition coefficient	*P*_art_	Blood/air	41.5	[9]
	*P*_liv_	Liver/blood	0.7	[9]
	*P*_rap_	Rapidly perfused/blood	0.7	Assumed to be same as liver
	*P*_kid_	Kidney/blood	0.7	Assumed to be same as liver
	*P*_slow_	Slowly perfused/blood	0.267	Muscle [9]
	*P*_adi_	Adipose/blood	7.8	[9]

Volume (L)	*V*_slow_	Volume fraction for slowly perfused	0.674	[11]
	*V*_liv_	Volume fraction for liver	0.044	[11]
	*V*_kidney_	Volume fraction for kidney	0.0075	[11]
	*V*_adi_	Volume fraction for adipose	0.112	[11]
	*V*_rap_	Rapidly perfused	0.91 − ∑*V*_*i*_	Total vol. — sum of all other
				compartments. Blood is 9% of BW

**Table 2:** Optimized parameter results.

Two-pathway model		Two-binding site model	
*V*_max⁡_	3.8 mg/hour	*V*_max_1__	3.7 mg/hour
*K*_*m*_	0.35 mg/liter	*K*_*m*_1__	0.3 mg/liter
*k*_GST_	4.5/hour	CL_2_	0.047 liter/hour

## Appendix

The differential equations used to describe each compartment in the PBPK model were developed using the law of conservation of mass: the amount of BCM flowing into each compartment must equal the amount of BCM leaving the compartment, whether it is through blood flow or ventilation, or through loss by metabolism or condensation. The following abbreviations can be found in Table 1:

(A.1) $$\begin{array}{cc} \frac{dA_{\text{ven}}}{dt} & =q_{1}C_{\text{rap}}+q_{2}C_{\text{adi}}+q_{3}C_{\text{slow}}\\ & \;+q_{4}C_{\text{kid}}+q_{5}C_{\text{liv}}\\ & \;-QCC\cdot C_{\text{ven}}, \end{array}$$

(A.2) $$\begin{array}{cc} \Rightarrow C_{\text{ven}} & =\frac{q_{1}C_{\text{rap}}+q_{2}C_{\text{adi}}+q_{3}C_{\text{slow}}+q_{4}C_{\text{kid}}+q_{5}C_{\text{liv}}}{QCC}, \end{array}$$

(A.3) $$\begin{array}{cc} \frac{dA_{\text{lung}}}{dt} & =QCC\cdot C_{\text{ven}}+QV\cdot C_{\text{inh}},\\ & \;-C_{\text{art}}\left(QCC+\left(\frac{QV}{P_{\text{Bld}}}\right)\right), \end{array}$$

(A.4) $$\begin{array}{cc} \Rightarrow C_{\text{art}} & =\frac{QCC\cdot C_{\text{ven}}+QV\cdot C_{\text{inh}}}{QCC+QV/P_{\text{Bld}}}, \end{array}$$

(A.5) $$\begin{array}{cc} C_{\text{exh}} & =\frac{C_{\text{art}}}{P_{\text{Bld}}}, \end{array}$$

(A.6) $$\begin{array}{cc} \frac{dA_{\text{inh}}}{dt} & =N\cdot QV\cdot \left(C_{\text{exh}}-C_{\text{inh}}\right)-\text{loss}\cdot A_{\text{inh}}, \end{array}$$

(A.7) $$\begin{array}{cc} \frac{dA_{\text{rap}}}{dt} & =q_{1}\cdot \left(C_{\text{art}\;}-C_{\text{rap}}\right) \end{array},$$

(A.8) $$\begin{array}{cc} \frac{dA_{\text{adi}}}{dt} & =q_{2}\cdot \left(C_{\text{art}}-C_{\text{adi}}\right), \end{array}$$

(A.9) $$\begin{array}{cc} \frac{dA_{\text{slow}}}{dt} & =q_{3}\cdot \left(C_{\text{art}}-C_{\text{slow}}\right) \end{array},$$

(A.10) $$\begin{array}{cc} \frac{dA_{\text{kid}}}{dt} & =q_{4}\cdot \left(C_{\text{art}}-C_{\text{kid}}\right)-Met_{K} \end{array},$$

(A.11) $$\begin{array}{cc} \frac{dA_{\text{liv}}}{dt} & =q_{5}\cdot \left(C_{\text{art}}-C_{\text{liv}}\right)-Met_{L}. \end{array}$$

For (A.1)–(A.11), we denote the venous concentration of BCM leaving a specific compartment comp as *C*_comp_ = *A*_comp_/(*V*_comp_ · *P*_comp_), where *A*_comp_ is the amount of BCM in the compartment, *V*_comp_ is the volume of the compartment, and *P*_comp_ is the partition coefficient for the compartment. Note there is no associated partition coefficient for the chamber, thus the concentration in the chamber is denoted by *C*_inh_ = *A*_inh_/*V*_cha_. Partition coefficients (given in Table 1) describe the measure of the differential solubility of BCM as the chemical passes between two different phases; this unitless ratio accounts for the amount of BCM absorbed by compartmental tissue as it passes from the compartment back into the bloodstream. Previous studies have determined the tissue/air and blood/air partition coefficients for BCM [9]. To determine the tissue/blood partition coefficient for each compartment, (A.12) is used:

(A.12) $$\begin{array}{cc} P_{\text{organ}} & =\text{tissue}∶\text{blood}=\frac{\text{tissue}∶\text{air}}{\text{blood}∶\text{air}}. \end{array}$$

Several assumptions were made for the construction of each equation in the system: Initially, a differential equation, (A.1), was created to represent the amount of BCM in the venous compartment. However, simulations in MatLab demonstrated that the amount of BCM in the venous compartment approached equilibrium very quickly with respect to the length of the experiment. This allowed us to set (A.1) equal to zero to obtain an algebraic expression for the concentration of BCM in the venous compartment with respect to the concentration of BCM in the other compartments. A similar technique was used with the lung compartment (A.3), obtaining an algebraic expression for the concentration of BCM in the arterial compartment.

## List of Abbreviations

**Chemicals**
4-MP: 4-methylpyrazole
BCM: Bromochloromethane
BDCM: Bromodichloromethane
DBP: Disinfection by-products
DCM: Dichloromethane
GST: Glutathione transferase
PBPK: Physiologically-based pharmacokinetic model
CCL: Candidate Contaminant List, Office of Water, US EPA.

**Equation Variables**
*V*_max⁡_: Maximum metabolic rate, mg/hour
*V*_max_*i*__: Maximum metabolic rate for binding site *i* in two-binding site hypothesis, mg/hour
*K*_*m*_: Affinity constant, mg/liter
*K*_*m*_*i*__: Affinity constant for binding site *i* in two-binding site hypothesis, mg/liter
*k*_GST_: Proportionality constant for linear pathway metabolized by GST, /hour
CL_2_: clearance constant consisting of the ratio of the maximum metabolic rate and the affinity constant, *V*_max_2__/*K*_*m*_2__, for second binding site, liter/hour
*A*_compartment_: Amount of BCM in compartment
*P*_compartment_: Partition coefficient for compartment
*V*_compartment_: Volume of compartment, liters
*C*_compartment_: Concentration of BCM in compartment, given by *C*_comp_ = *A*_comp_/(*V*_comp_ · *P*_comp_).

## References

[1] U.S. EPA (2009). Bromochloromethane testing rationale.

[2] U.S. EPA (1990). Health and environmental effects document for bromochloromethane.

[3] U.S. EPA (2002). The occurrence of disinfection by-products (dbps) of health concern in drinking water: results of a nationwide dbp occurrence study.

[4] Richardson SD, Plewa MJ, Wagner ED, Schoeny R, DeMarini DM (2007). Occurrence, genotoxicity, and carcinogenicity of regulated and emerging disinfection by-products in drinking water: a review and roadmap for research. *Mutation Research*.

[5] Wei YY, Liu Y, Dai RH (2011). Trihalomethanes and haloacetic acid species from the chlorination of algal organic matter and bromide. *Water Science and Technology*.

[6] Thompson CM, Sonawane B, Barton HA (2008). Approaches for applications of physiologically based pharmacokinetic models in risk assessment. *Journal of Toxicology and Environmental Health B*.

[7] Edginton AN, Joshi G (2011). Have physiologically-based pharmacokinetic models delivered?. *Expert Opinion on Drug Metabolism and Toxicology*.

[8] Gargas ML, Andersen ME (1989). Determining kinetic constants of chlorinated ethane metabolism in the rat from rates of exhalation. *Toxicology and Applied Pharmacology*.

[9] Gargas ML, Andersen ME, Clewell HJ (1986). A physiologically based simulation approach for determining metabolic constants from gas uptake data. *Toxicology and Applied Pharmacology*.

[10] Brown RP, Delp MD, Lindstedt SL, Rhomberg LR, Beliles RP (1997). Physiological parameter values for physiologically based pharmacokinetic models. *Toxicology and Industrial Health*.

[11] Delp MD, Evans MV, Duan C (1998). Effects of aging on cardiac output, regional blood flow, and body composition in Fischer-344 rats. *Journal of Applied Physiology*.

[12] Medinsky MA, Sabourin PJ, Henderson RF, Lucier G, Birnbaum LS (1989). Differences in the pathways for metabolism of benzene in rats and mice simulated by a physiological model. *Environmental Health Perspectives*.

[13] HSE (2000). *Bromochloromethane Risk Assessment Document*.

[14] Collom SL, Laddusaw RM, Burch AM, Kuzmic P, Perry MD, Miller GP (2008). CYP2E1 substrate inhibition: mechanistic interpretation through an effector site for monocyclic compounds. *Journal of Biological Chemistry*.

[15] Anders MW (2008). Chemical toxicology of reactive intermediates formed by the glutathione-dependent bioactivation of halogen-containing compounds. *Chemical Research in Toxicology*.

[16] Thier R, Taylor JB, Pemble SE (1993). Expression of mammalian glutathione S-transferase 5-5 in Salmonella typhimurium TA1535 leads to base-pair mutations upon exposure to dihalomethanes. *Proceedings of the National Academy of Sciences of the United States of America*.

[17] Kundu B, Richardson SD, Granville CA (2004). Comparative mutagenicity of halomethanes and halonitromethanes in Salmonella TA100: structure-activity analysis and mutation spectra. *Mutation Research*.

[18] White RD, Gandolfi AJ, Bowden GT, Sipes IG (1983). Deuterium isotope effect on the metabolism and toxicity of 1,2-dibromoethane. *Toxicology and Applied Pharmacology*.

[19] Tracy TS (2006). Atypical cytochrome P450 kinetics: implications for drug discovery. *Drugs in R & D*.

[20] Korzekwa KR, Krishnamachary N, Shou M (1998). Evaluation of atypical cytochrome P450 kinetics with two-substrate models: evidence that multiple substrates can simultaneously bind to cytochrome P450 active sites. *Biochemistry*.

[21] Evans MV, Crank WD, Yang HM, Simmons JE (1994). Applications of sensitivity analysis to a physiologically based pharmacokinetic model for carbon tetrachloride in rats. *Toxicology and Applied Pharmacology*.

[22] McNally K, Cotton R, Loizou GD (2011). A workflow for global sensitivity analysis of PBPK models. *Frontiers in Pharmacology*.

[23] Ramsey JC, Andersen ME (1984). A physiologically based description of the inhalation pharmacokinetics of styrene in rats and humans. *Toxicology and Applied Pharmacology*.

[24] Evans MV, Caldwell JC (2010). Evaluation of two different metabolic hypotheses for dichloromethane toxicity using physiologically based pharmacokinetic modeling for in vivo inhalation gas uptake data exposure in female B6C3F1 mice. *Toxicology and Applied Pharmacology*.

[25] Corley RA, Mendrala AL, Smith FA (1990). Development of a physiologically based pharmacokinetic model for chloroform. *Toxicology and Applied Pharmacology*.

[26] Chiu WA, Okino MS, Evans MV (2009). Characterizing uncertainty and population variability in the toxicokinetics of trichloroethylene and metabolites in mice, rats, and humans using an updated database, physiologically based pharmacokinetic (PBPK) model, and Bayesian approach. *Toxicology and Applied Pharmacology*.

[27] Evans MV, Chiu WA, Okino MS, Caldwell JC (2009). Development of an updated PBPK model for trichloroethylene and metabolites in mice, and its application to discern the role of oxidative metabolism in TCE-induced hepatomegaly. *Toxicology and Applied Pharmacology*.

[28] Andersen ME, Clewell HJ, Gargas ML, Smith FA, Reitz RH (1987). Physiologically based pharmacokinetics and the risk assessment process for methylene chloride. *Toxicology and Applied Pharmacology*.

[29] Jepson GW, McDougal JN (1997). Physiologically based modeling of nonsteady state dermal absorption of halogenated methanes from an aqueous solution. *Toxicology and Applied Pharmacology*.

[30] Jepson GW, McDougal JN (1999). Predicting vehicle effects on the dermal absorption of halogenated methanes using physiologically based modeling. *Toxicological Sciences*.

[31] Johnson MK (1966). Studies on glutathione S-alkyltransferase of the rat. *Biochemical Journal*.

[32] Pankow D, Weise M, Hoffmann P (1992). Effect of isoniazid or phenobarbital pretreatment on the metabolism of dihalomethanes to carbon monoxide. *Polish Journal of Occupational Medicine and Environmental Health*.

[33] Li J, Wei DQ, Wang JF, Li YX (2011). A negative cooperativity mechanism of human CYP2E1 inferred from molecular dynamics simulations and free energy calculations. *Journal of Chemical Information and Modeling*.

[34] Dey A, Kumar SM (2011). Cytochrome P450 2E1 and hyperglycemia-induced liver injury. *Cell Biology and Toxicology*.

[35] Porubsky PR, Meneely KM, Scott EE (2008). Structures of human cytochrome P-450 2E1: insights into the binding of inhibitors and both small molecular weight and fatty acid substrates. *Journal of Biological Chemistry*.

[36] Cobelli C, DiStefano JJ (1980). Parameter and structural identifiability concepts and ambiguities: a critical review and analysis. *American Journal of Physiology*.

[37] Kim C, Manning RO, Brown RP, Bruckner JV (1996). Use of the vial equilibration technique for determination of metabolic rate constants for dichloromethane. *Toxicology and Applied Pharmacology*.

[38] Feierman DE, Cederbaum AI (1986). Inhibition of microsomal oxidation of ethanol by pyrazole and 4-methylpyrazole in vitro. Increased effectiveness after induction by pyrazole and 4-methylpyrazole. *Biochemical Journal*.

[39] Koop DR (1986). Hydroxylation of p-nitrophenol by rabbit ethanol-inducible cytochrome P-450 isozyme 3a. *Molecular Pharmacology*.

